# The influence of digital addiction on adolescents’ subjective wellbeing: a meta-analysis

**DOI:** 10.3389/fpsyg.2026.1776619

**Published:** 2026-03-05

**Authors:** Jinxin Zhou, Ziyue Chen, Huiling Wu, Guoxiang Guan, Yijing Li, Yanru Kang, Runjie Sun, Yongbing Liu

**Affiliations:** School of Nursing, Faculty of Medicine, Yangzhou University, Yangzhou, China

**Keywords:** adolescents, digital addiction, mental health, meta-analysis, subjective wellbeing

## Abstract

**Objective:**

This study aims to quantitatively and comprehensively evaluate the association between digital addiction (DA) and subjective well-being (SWB) in adolescents.

**Methods:**

A comprehensive search was conducted across multiple electronic databases, including PubMed, Web of Science, Embase, EBSCO, Cochrane Library, CNKI, Wanfang, and VIP, to identify studies examining the correlation between DA and SWB in adolescents. Subgroup and sensitivity analyses were conducted to explore sources of heterogeneity and assess the robustness of the pooled estimates. Publication bias was evaluated using funnel plots, Begg’s and Egger’s tests, the PET–PEESE approach, and the trim-and-fill method.

**Results:**

This study was registered in PROSPERO (registration number: CRD420251014128). The meta-analysis included 17 studies comprising a total of 30,915 adolescents. A random-effects model was employed for the pooled analysis, which revealed a significant negative correlation between DA and SWB (Fisher’s *Z* = –0.30, 95% CI [−0.34, −0.27]). A high level of heterogeneity was observed (*I*^2^ = 86.6%). Subgroup analyses indicated that the association between DA and SWB was moderated by region and study quality, but not by sample size, publication year, DA subtype, or participant age.

**Conclusion:**

DA is negatively correlated with SWB. Therefore, it is essential to establish a comprehensive intervention framework that integrates family, school, societal, and individual efforts. This can be accomplished through transforming parenting practices within families, enhancing digital health literacy and psychological screening in schools, strengthening online supervision and accountability in society, and fostering self-directed learning skills among adolescents, ultimately improving their SWB.

**Systematic review registration:**

https://www.crd.york.ac.uk/prospero/display_record.php?ID=CRD420251014128, identifier PROSPERO (CRD420251014128).

## 1 Introduction

In the 21st century, marked by a profound digital revolution, the rapid proliferation and integration of digital devices into adolescents’ daily lives have occurred at an unprecedented rate (Ding and Li, 2023). However, excessive engagement in digital activities has been shown to exert substantial negative effects on physical and mental health, academic achievement, occupational functioning, and social skills, potentially leading to DA (Christakis, 2019). At the same time, research on digital platforms has shown that technology design, perceived credibility, and user experience jointly shape individuals’ behavioral intentions in online environments (Wang et al., 2024), highlighting that adolescents’ engagement with digital media is embedded in broader sociotechnical systems rather than being a purely individual choice. The concept of DA has attracted increasing scholarly attention; however, its definition and theoretical framework remain contested and require substantial refinement. At present, no consensus exists within the academic community regarding the criteria for distinguishing excessive use of digital technologies from a state of addiction. Studies employing diverse measurement dimensions and assessment methods frequently produce markedly divergent findings. This definitional ambiguity substantially hampers the advancement of research on DA. Generally, digital addiction is an umbrella term that refers to a range of problematic behaviors stemming from the excessive use of digital media (Basel et al., 2020), encompassing subtypes such as internet addiction (IA), gaming addiction (GA), smartphone addiction (SA), and social media addiction (SMA) (Christakis, 2019). A meta-analysis encompassing data from 64 countries reported estimated global prevalence rates of 14.22% for internet addiction, 6.04% for gaming addiction, 26.99% for smartphone addiction, and 17.42% for social media addiction (Meng et al., 2022). Compared to the general population, adolescents are more vulnerable to the influence of digital technologies, which exacerbates their risk of developing mental health problems (Cai et al., 2023; Dienlin and Johannes, 2022).

Digital addiction has been associated with a range of adverse psychological outcomes, including anxiety, depression, suicidal ideation, and reduced subjective well-being (Hayashi et al., 2017; Mortazavi et al., 2023; Shakya and Christakis, 2017). Adolescence represents a critical developmental stage, marked by profound physical, cognitive, and social transformations, making SWB a central construct for understanding adolescent health and development (Taghipour et al., 2023). SWB refers to an individual’s overall evaluation of their quality of life and comprises two dimensions: cognitive appraisal (life satisfaction) and emotional experiences (both positive and negative) (Diener, 2000). It is considered a key indicator of an individual’s mental health (Cao et al., 2025). Studies have shown that adolescents with high levels of DA tend to report the lowest levels of SWB, experience difficulties in maintaining interpersonal relationships, exhibit escapist behaviors, and possess a negative self-concept (Ding et al., 2024). During adolescence, individuals are particularly vulnerable to fluctuations in well-being arising from the interplay of internal and external factors, including mental health, self-perceived health, and social relationships (Klinger et al., 2024; Zhang et al., 2023). Empirical studies indicate that dimensions of self-perceived health—such as general health, vitality, and mental health—are positively associated with overall SWB, with mental health emerging as a particularly strong predictor (Matic and Musil, 2023). These findings underscore the central role of psychological and health-related factors in shaping adolescent well-being and highlight the need for targeted interventions to strengthen them (Buchanan et al., 2022). Beyond self-report and cross-sectional survey designs, emerging work has started to leverage machine-learning approaches to automatically detect mental disorders based on individuals’ digital traces (Hussain, 2024), further underscoring the central role of digital environments in contemporary mental-health research.

Moreover, negative well-being during adolescence can exert profound long-term consequences, influencing mental health, social relationships, and career attainment in adulthood (Waller et al., 2023). Positive emotions in early adolescence have been found to predict lower levels of depression, anxiety, and loneliness in early adulthood, alongside higher career satisfaction and self-worth (Belen, 2024). Conversely, persistently low levels of SWB in adolescence are linked to heightened vulnerability to mental health disorders and compromised social functioning (Park et al., 2023). This is particularly concerning given the high prevalence of depressive and anxiety symptoms among adolescents, with evidence indicating that these symptoms reach clinical thresholds in a considerable proportion of this population (Gurven et al., 2024). The enduring consequences of negative well-being underscore the importance of early interventions and the cultivation of positive psychological constructs during adolescence (Wen et al., 2022). In conclusion, subjective well-being in adolescence is a multidimensional construct shaped by the dynamic interplay of psychological, health-related, and environmental factors. Adolescents’ vulnerability to fluctuations in well-being, coupled with the enduring consequences of negative well-being, underscores the necessity of implementing early interventions and fostering positive psychological development.

According to Self-Determination Theory, individuals have three basic psychological needs: autonomy, competence, and relatedness (Ryan and Deci, 2022). However, DA often impairs the fulfillment of these needs. DA, conceptualized as an “embodied habit,” shapes behavior through repetitive online engagement, creating a false sense of autonomy that diminishes self-reflective regulation and contributes to identity confusion, thereby undermining the need for autonomy. The “substituted sense of competence” formed in the virtual environment is often difficult to transfer to real-life situations, and repeated failures in offline tasks diminish authentic self-efficacy, thereby compromising the need for competence. Moreover, the superficiality of virtual social interactions cannot replace authentic emotional bonds, often resulting in feelings of loneliness and social alienation, which frustrate the need for relatedness. The interplay among these unmet needs forms a self-reinforcing cycle: “digital addiction → psychological need deprivation → decline in SWB” (Schlimme, 2010). In line with Social Support Theory, DA may disrupt adolescents’ existing social support systems, reducing emotional support from family, school, and peers, and consequently diminishing their SWB (Sarason, 2013; Vaux, 1988).

Although numerous studies in recent years have examined the relationship between DA and SWB among adolescents, most have focused on individual subtypes of DA (e.g., internet addiction, gaming addiction, smartphone addiction, or social media addiction) rather than considering DA as a unified construct. Furthermore, existing studies on SWB tend to examine only a single dimension of the construct. For instance, Cao et al. investigated the association between internet addiction and life satisfaction (Cao et al., 2011), whereas Fabris et al. (2020) focused on the relationship between social media addiction and emotional experiences. At the evidence-synthesis level, prior meta-analyses have primarily concentrated on the associations between problematic internet use and general mental health outcomes such as depression, anxiety, and stress, or on specific psychosocial correlates such as social support, without providing a quantitative summary of the link between DA and adolescents’ multidimensional SWB. In addition, the extent to which study-level characteristics (e.g., cultural context, DA subtype, measurement instruments, and age group) account for between-study variability in effect sizes remains unclear.

Building on this body of work, the present pre-registered meta-analysis addresses these gaps in three ways. First, it treats DA as an overarching construct that integrates different digital addiction subtypes while still distinguishing them analytically. Second, it synthesizes the available empirical evidence on the association between DA and adolescents’ SWB and estimates a pooled effect size for this relationship. Third, through subgroup analyses and meta-regression, it systematically evaluates whether key study-level characteristics—such as geographic region, DA subtype, study quality, and age—moderate the DA–SWB association. In doing so, this study aims to deepen the theoretical understanding of how DA is linked to adolescent SWB and to provide evidence-based implications for preventing and mitigating the negative impact of DA on adolescents’ mental health.

Building on the current state of research, this study integrates existing findings on the relationship between DA and SWB in adolescents and performs a meta-analysis to assess whether adolescents with DA are at higher risk for reduced SWB. Specifically, the primary objective of this study is to examine the direction of the correlation and calculate the overall effect size between DA and SWB. This study aims to deepen the theoretical understanding of the relationship between DA and adolescent SWB, while also providing insights to prevent and mitigate the negative effects of DA on adolescents’ mental health.

The present meta-analysis makes several contributions to the literature on DA and adolescent well-being. First, unlike previous work that has mainly focused on single subtypes of DA (e.g., internet, gaming, smartphone, or social media addiction) or on one narrow component of SWB, we synthesize evidence across multiple DA subtypes and both cognitive (life satisfaction) and affective components of SWB in adolescents. Second, by leveraging a preregistered protocol, a comprehensive search of international and Chinese databases, and standardized meta-analytic techniques, we provide the most up-to-date and quantitative estimate of the overall association between DA and SWB in youth. Third, we systematically examine potential moderators, including geographic region, DA subtype, age group, and study quality, thereby identifying contextual and methodological conditions under which the DA–SWB association may vary. Finally, the findings offer an integrative framework for designing multi-level interventions in families, schools, and communities to buffer the adverse impact of DA on adolescents’ subjective well-being. In contrast to earlier studies that have typically examined digital behaviors in isolation or focused on specific outcomes such as mental disorders or user intentions, our work centers on subjective well-being as an integrative indicator of youth mental health and explicitly models DA as an umbrella construct spanning multiple digital contexts.

## 2 Method

### 2.1 Literature search

This meta-analysis was performed in accordance with the 2020 Preferred Reporting Items for Systematic Reviews and Meta-Analyses (PRISMA) guidelines (Page et al., 2021). Additionally, the protocol was registered with the international prospective systematic review registry PROSPERO (CRD420251014128). Research on the relationship between DA and SWB, published up to April 10, 2025, was retrieved from eight databases: PubMed, Web of Science, Embase, EBSCO, Cochrane Library, China National Knowledge Infrastructure (CNKI), Wanfang, and Chongqing VIP Information Co., Ltd. (VIP). To minimize the risk of overlooking relevant articles, the search strategy incorporated both Medical Subject Headings (MeSH) and free-text terms. The search terms included: adolescents, digital addiction, internet addiction, online gaming addiction, smartphone addiction, social media addiction, and subjective well-being.

### 2.2 Inclusion and exclusion criteria

Inclusion Criteria: Studies meeting the following criteria will be included.

(1) Original research published in English or Chinese; (2) Must report on the relationship between DA and SWB; (3) Participants should be adolescents aged 10–25 [Arnett divided adolescents into traditional adolescence (aged 10–18 years) and emerging adulthood (aged 18–25 years)] (Arnett, 2001), excluding individuals who are prisoners or patients; (4) Must report the sample size; (5) Should employ validated scales to assess DA and SWB; (6) Must report the Pearson correlation coefficient (*r*).

Exclusion Criteria: Studies meeting the following criteria will be excluded.

(1) Non-empirical research (including reviews, commentaries, letters to the editor, and conference abstracts); (2) Books; (3) Dissertations; (4) Exposures or outcomes that do not align with the definitions of DA or SWB; (5) Missing relevant dimensions to SWB; (6) Lack of relevant outcome indicators.

**Figure 1:**
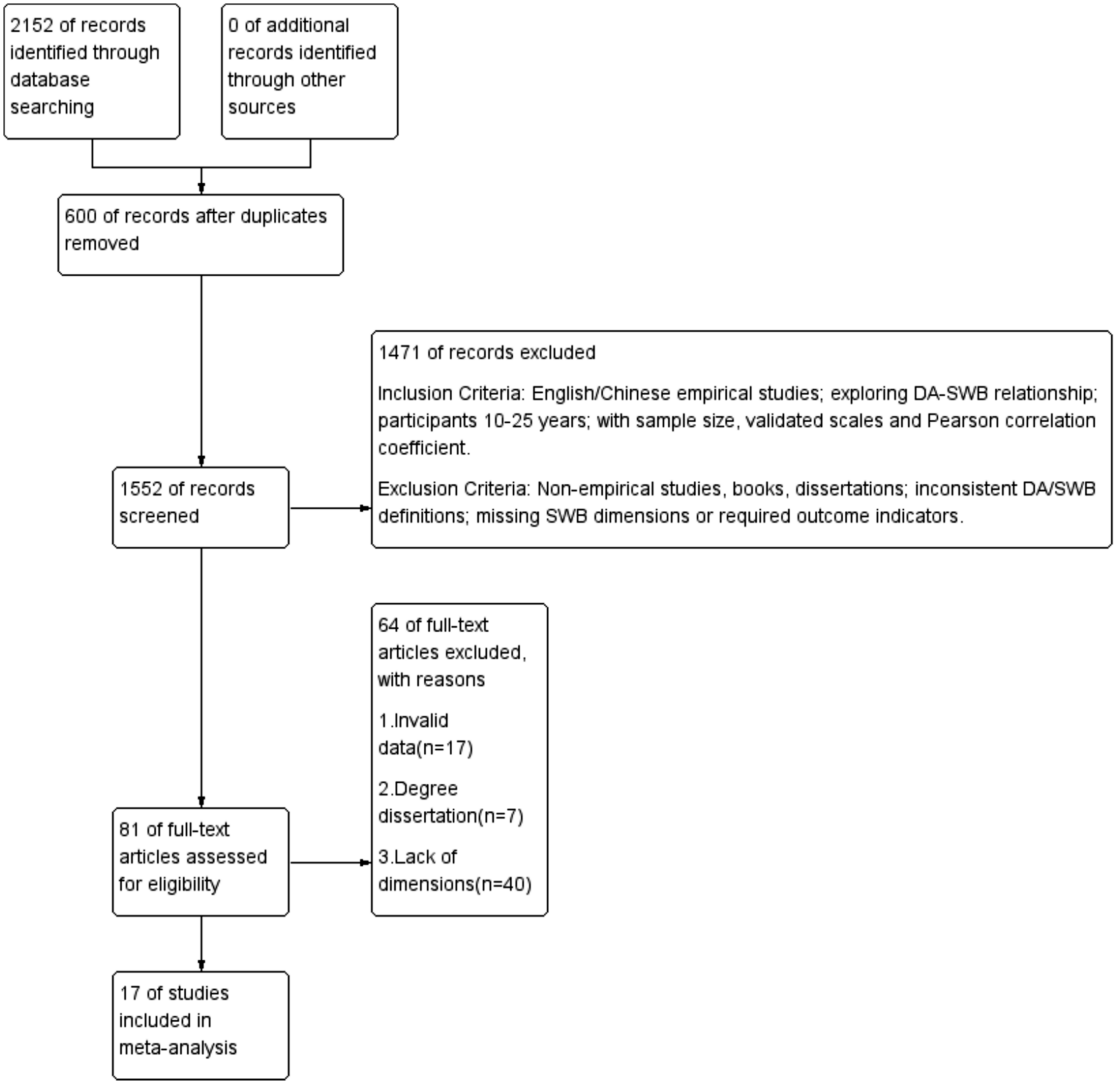
Flow diagram.

As shown in Figure 1, the flow diagram is presented. A detailed description of the search strategy is provided in the Appendix.

### 2.3 Quality evaluation

The quality of the included studies was independently evaluated by two reviewers. Methodological quality was assessed using the JBI Critical Appraisal Checklist for Analytical Cross-Sectional Studies (Aromataris et al., 2024), and detailed results are provided in the Appendix. In accordance with the aforementioned inclusion criteria, relevant data were extracted from the eligible full-text articles, including author names, year of publication, country, sample size (by gender), age, study design, measurement instruments for DA and SWB, and Pearson correlation coefficients (r values).

### 2.4 Effect size calculation

Effect sizes were derived from the *r* values reported in the included studies. Specifically, Fisher’s *Z* transformation was applied, with weights based on sample size and corresponding 95% confidence intervals (Lei et al., 2018):


$$Z=0.5\times \frac{\ln1+r}{\ln1-r}$$

The variance of *Z* was calculated as:


$$V_{Z}=\frac{1}{n-3}$$

And the standard error of *Z* is given by:


$$S_{E}=\sqrt{V_{Z}}.$$

When a study reported multiple DA-SWB correlations based on different SWB subscales (e.g., life satisfaction and affective well-being) or multiple DA measures within the same sample, we ensured that each independent sample contributed only one effect size to the meta-analysis. This approach avoids violating the independence assumption while preserving information from multiple relevant indicators. When clearly independent sub-samples were reported (e.g., separate school cohorts), we treated them as separate studies in the analysis.

### 2.5 Data processing and analysis

The analysis was conducted using STATA MP 18 statistical software. The r values from the included studies were transformed into Fisher’s *Z* values using the specified formula, and both the Fisher’s *Z* values and their standard errors ( $S_{E}$ ) were input into STATA MP 18. The *I*^2^ statistic was used to assess the heterogeneity of the included studies. *I*^2^ > 50% or *p* < 0.05 typically indicates significant statistical heterogeneity, and a random effects model was used to calculate the effect size. Otherwise, a fixed effects model was employed (Barili et al., 2018). Subgroup analyses were conducted based on sample size, publication year, country, age, study quality, and DA subtype. Given the limited number of included studies (*k* = 17), formal meta-regression was not performed to avoid unstable estimates and overfitting. Sensitivity analyses were performed by sequentially excluding individual studies to assess the robustness of the findings. In addition, we performed a sensitivity analysis to correct for attenuation due to measurement unreliability by re-estimating the pooled effect under a conservative assumption that both DA and SWB measures had reliabilities of 0.70. Publication bias was assessed and robustness was examined using funnel plots, Begg’s test, Egger’s test, and the PET-PEESE approach (Precision-Effect Test/Precision-Effect Estimate with Standard Error). If potential publication bias was identified, the trim-and-fill method was applied to recalculate the adjusted combined effect size.

## 3 Results

### 3.1 Characteristics of included studies

A total of 2,152 articles were retrieved from eight databases, ultimately resulting in 17 studies. All studies included in this meta-analysis were cross-sectional and involved 30,915 adolescents, with sample sizes ranging from 100 to 11,401. The studies were conducted in six countries: China (*n* = 11), Turkey (*n* = 2), India (*n* = 1), Bangladesh (*n* = 1), Palestine (*n* = 1), and Iran (*n* = 1). A summary of the characteristics of the included studies is provided in Table 1.

**Table 1:** Characteristics of 17 studies included in the meta-analysis.

Study	Country	Sample size (male/female)	Age (Mean ± SD or age range)	Design	DA measure	SWB measure	Main findings
Afroz (2016)	India	100 (50/50)	18–23	Cross-sectional	Internet addiction test (IAT)	Subjective wellbeing inventory (SUBI)	−0.28
Cheng and Lin (2023)	China	1,043 (533/510)	14.98 ± 1.55	Cross-sectional	Compulsive internet use scale	Psychological wellbeing subscale of the kidscreen-52	−0.311
Ding et al. (2024)	China	694 (311/383)	18–25	Cross-sectional	Short video addiction scale (SVAS)	Subjective wellbeing scale	−0.16
Huang et al. (2023)	China	668 (373/295)	18–25	Cross-sectional	Mobile phone addiction index (MPAI)	Subjective wellbeing questionnaire (SWQ)	−0.28
Islam et al. (2025)	Bangladesh	384 (195/189)	18.99 ± 1.50	Cross-sectional	Smartphone addiction scale-short version (SAS-SV)	Subjective happiness scale (SHS)	−0.26
Li et al. (2021)	China	940 (467/473)	12–16	Cross-sectional	Smartphone addiction index (SAI)	General wellbeing schedule (GWB)	−0.393
Li et al. (2022)	China	2,627 (1,268/1359)	14.42 ± 1.55	Cross-sectional	Chinese version of pathological online game use questionnaire (POGU)	Satisfaction with Life Scale (SWLS); Positive and negative emotions scale	−0.261
Lin et al. (2023)	China	1,060	14.66 ± 0.86	Cross-sectional	Chinese internet addiction scale-revised	Chinese happiness inventory	−0.39
Liu et al. (2024)	China	7,669 (2,999/4670)	18–25	Cross-sectional	Problematic Internet use questionnaire (PIUQ-SF6)	WHO five-item wellbeing index	−0.211
Mahmid et al. (2021)	Palestine	566 (306/260)	18–25	cross-sectional	Problematic internet usage scale (IDS9-SF)	Scale of general wellbeing (SGWB)	−0.32
Mei et al. (2015)	China	1,551 (651/900)	15.42 ± 1.92	Cross-sectional	Young’s diagnostic questionnaire for internet addiction	Index of wellbeing scale	−0.26
Odaci and Cikrikci (2014)	Turkey	380 (152/228)	19.61 ± 1.12	Cross-sectional	Problematic internet use scale	Subjective wellbeing scale	−0.38
Rastegarian et al. (2022)	Iran	500 (0/500)	16.7 ± 0.97	Cross-sectional	Young’s diagnostic questionnaire for internet addiction	Oxford happiness questionnaire (OHQ)	−0.339
Sun et al. (2022)	China	518 (143/375)	18–25	Cross-sectional	Revised Chen internet addiction scale (CIAS-R)	Subjective wellbeing scale	−0.279
Uysal et al. (2013)	Turkey	297 (140/157)	20.1 ± 1.3	Cross-sectional	Bergen Facebook addiction scale (BFAS)	Subjective happiness scale	−0.37
Ye et al. (2022)	China	517 (222/295)	19.19 ± 1.12	Cross-sectional	Adapted the game addiction scale	Very short version of the Chinese wellbeing scale	−0.33
Zhou et al. (2022)	China	11,401 (5,649/5752)	10–11	Cross-sectional	Smartphone addiction proneness scale for youth	Index of wellbeing (IWB)	−0.21

### 3.2 Data analysis

**Figure 2:**
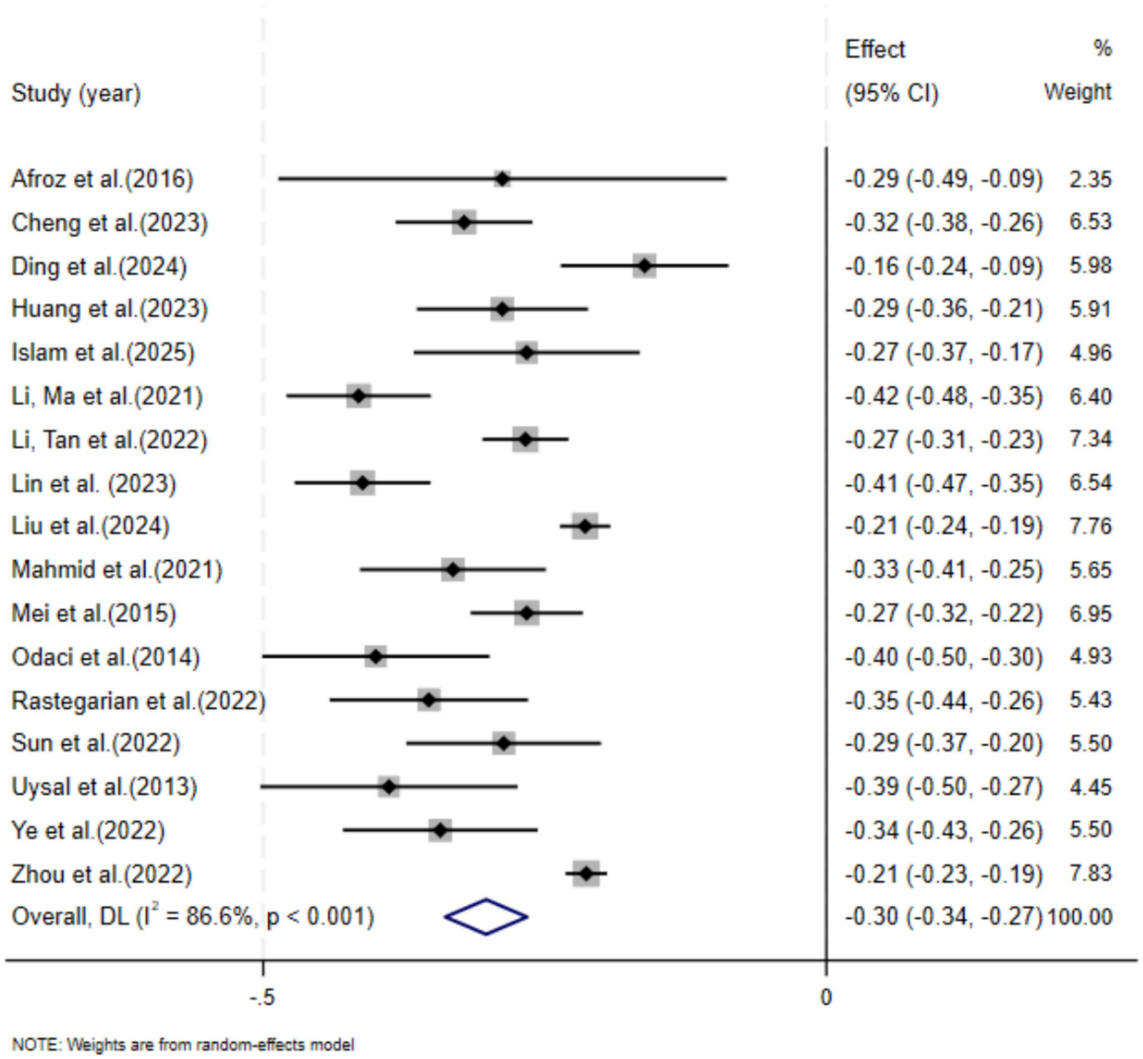
Forest plot of the relationship between DA and SWB in adolescents.

Figure 2 presents the results of the meta-analysis. The analysis revealed a pooled effect size of −0.30 (95% CI [−0.34, −0.27]), indicating a significant negative association between DA and SWB. However, substantial heterogeneity (*I*^2^ = 86.6%, *p* < 0.001) was observed among the included studies.

Given the substantial heterogeneity (*I*^2^ = 86.6%), we have now reported the prediction interval (PI) under the random-effects model to reflect the expected range of true effects in a future comparable study. Specifically, we estimated the between-study variance (τ^2^) using the DerSimonian-Laird (DL) estimator, consistent with our primary random-effects specification in Stata, and we did not apply the Hartung-Knapp (HK/HKSJ) adjustment. Under the DL random-effects model, the pooled effect size was −0.30 (95% CI [−0.34, −0.27]), and the corresponding 95% prediction interval was [−0.45, −0.16]. These results have been added to the Results section (heterogeneity/random-effects results) and the statistical analysis description has been updated accordingly.

### 3.3 Subgroup analysis

To further explore the potential sources of heterogeneity, subgroup analyses were performed based on sample size, geographical location, study quality, types of digital addiction, publication year, and age. The results, presented in Table 2, suggest that geographical location and study quality may contribute to the observed heterogeneity. Studies conducted in countries other than China reported a *Z* value of −0.34 (95% CI [−0.38, −0.30]), indicating low heterogeneity (*I*^2^ = 0%, *p* = 0.478), whereas studies conducted in China had a *Z* value of −0.29 (95% CI [−0.33, −0.25]) with *I*^2^ = 89.5% (*p* < 0.001). For studies with a JBI score of 4–6, the *Z* value was −0.31 (95% CI [−0.37, −0.25]), indicating lower heterogeneity (*I*^2^ = 35.1%, *p* = 0.202), whereas studies with a JBI score of 7–8 reported a *Z* value of −0.30 (95% CI [−0.34, −0.26]) with *I*^2^ = 89% (*p* < 0.001).

**Table 2:** Results of subgroup analysis on the relationship between DA and SWB in adolescents.

Subgroup characteristics	*k*	Fisher’s *Z* value (95% CI)	*I*^2^ (%)	*p-*value
Sample size				
≥1,000	6	−0.29 (−0.34,–0.23)	91.1	<0.001
<1,000	11	−0.31 (−0.36,–0.26)	73.4	<0.001
Geographic location				
China	11	−0.29 (−0.33,–0.25)	89.5	<0.001
Other countries	6	−0.34 (−0.38,−0.30)	0	0.478
Quality				
7–8	12	–0.30 (−0.34,–0.26)	89	<0.001
4–6	5	−0.31 (−0.37,–0.25)	35.1	0.202
Types of DA				
IA	9	−0.32 (−0.37,–0.26)	86.5	<0.001
GA	2	−0.29 (−0.37,–0.22)	59.3	0.117
SA	4	−0.29 (−0.40,–0.19)	92.1	<0.001
SMA	2	−0.27 (−0.49,–0.05)	90.5	0.001
Year of publication				
2013–2019	4	−0.33 (−0.42,-0.25)	61.7	0.05
2020–2025	13	−0.29 (−0.34,-0.25)	88.5	<0.001
Age				
<18	7	−0.32 (−0.38,-0.25)	92.5	<0.001
≥18	10	−0.29 (−0.34,-0.27)	76.8	<0.001

k: Number of included studies; DA: Digital addiction; IA: Internet addiction; GA: Gaming addiction; SA: Smartphone addiction; SMA: Social media addiction.

### 3.4 Sensitivity analysis

**Figure 3:**
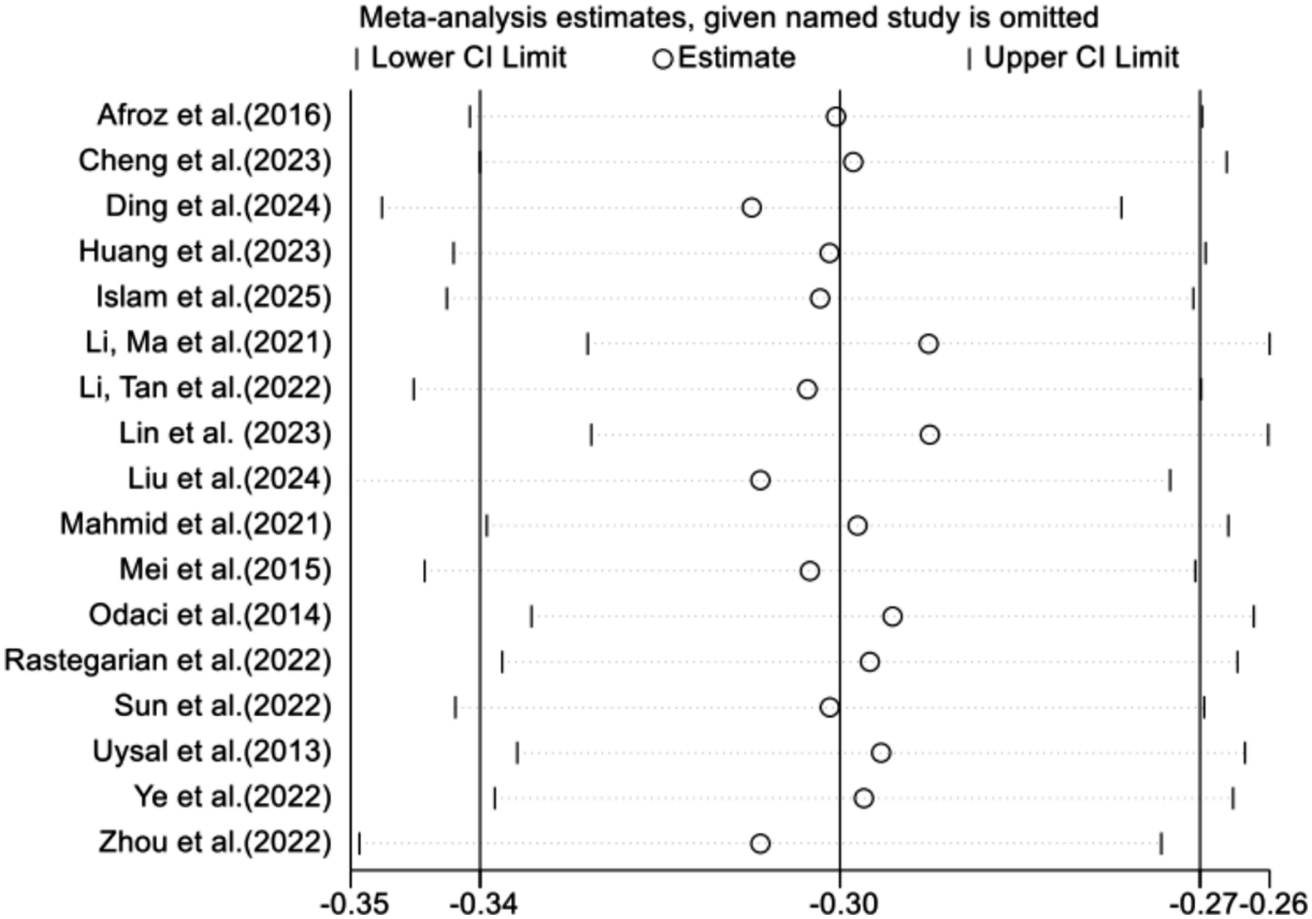
Sensitivity analysis of DA and SWB.

A sensitivity analysis was conducted to assess the influence of individual studies on the overall pooled effect size. As shown in Figure 3, the pooled effect sizes, after excluding each study, were primarily centered around −0.30, with confidence intervals remaining stable. This suggests that individual studies have limited impact on the overall results, indicating that the findings are relatively robust.

As a robustness check, we conducted a sensitivity analysis correcting for attenuation due to measurement unreliability. Specifically, we first transformed the *Fisher’s Z* effect size reported in each study back to a correlation coefficient (*r* = tanh(*z*)). Assuming a conservative scenario in which both the DA and SWB measures had reliabilities of 0.70 (i.e., *ρ*X = *ρ*Y = 0.70), we adjusted the observed correlations for reliability-induced attenuation and then re-transformed the corrected correlations to *Fisher’s Z* (z_true_ = atanh(r_true_)). Under this conservative reliability assumption, the pooled effect size was *z* = −0.36 (SE = 0.00875, 95% CI [−0.377, −0.343]), corresponding to *r* = −0.34 (95% CI [−0.360, −0.330]). The effect remained statistically significant and of similar magnitude, indicating that the main conclusion of a moderate negative association between DA and adolescents’ SWB is robust even when accounting for potential attenuation due to measurement error.

### 3.5 Publication bias

**Figure 4:**
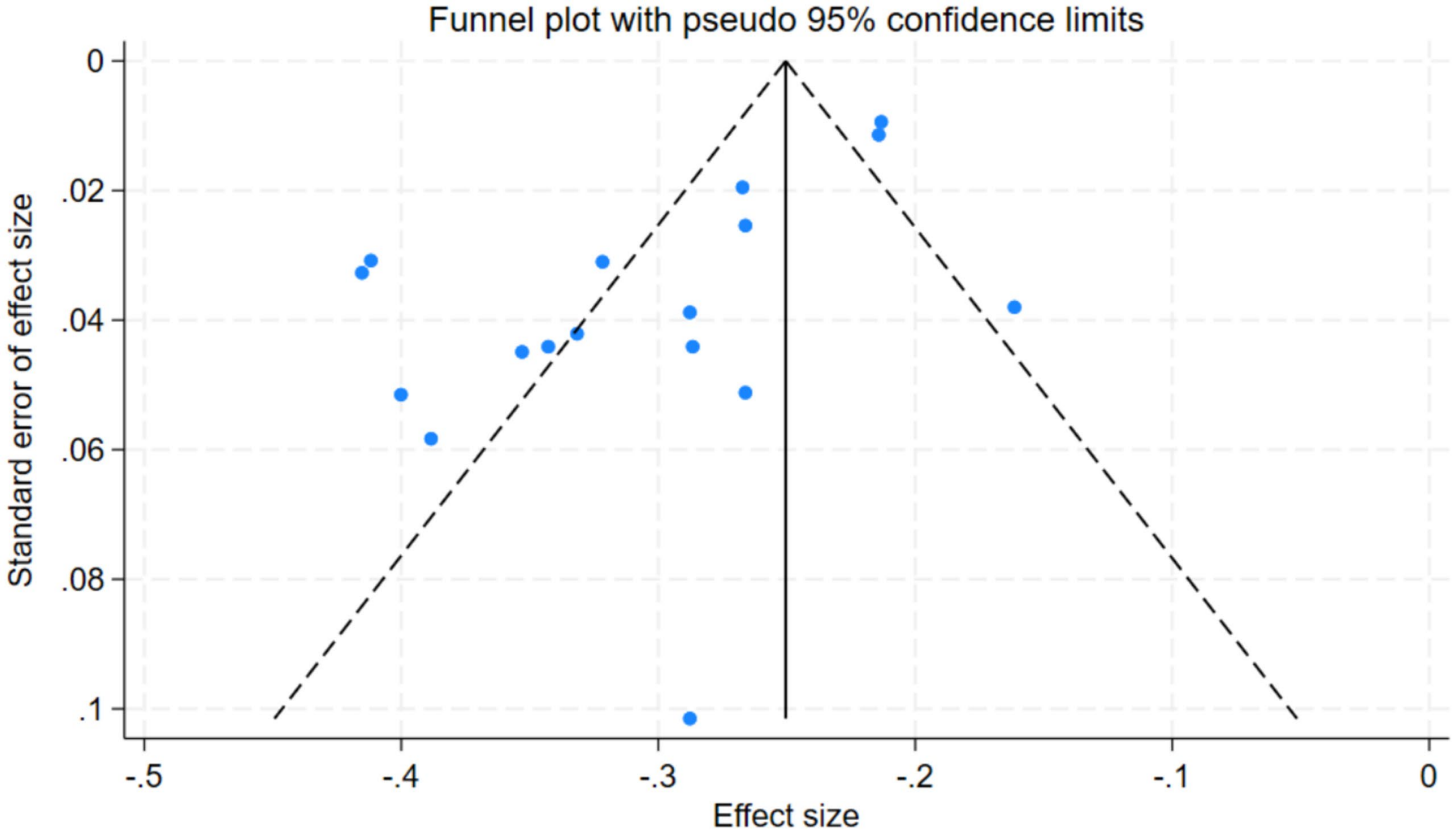
Funnel plot of DA and SWB.

Publication bias was assessed using a funnel plot, Begg’s test and Egger’s test. As shown in the funnel plot (Figure 4), the effect sizes were asymmetrically distributed, and Begg’s test (*p* < 0.001) and Egger’s test (*p* < 0.001) indicated significant publication bias. Meanwhile, the PET-PEESE test (*p* < 0.001) yielded significant results, further supporting the reliability of the publication-bias assessment conclusions. These tests are all reasonable and valid robustness checks. Therefore, the trim-and-fill method was applied. The adjusted effect size showed no substantial change, and no missing studies were imputed, indicating that the overall findings remained statistically significant and robust. These findings suggest that publication bias did not substantially affect the validity of the results, and multiple robustness checks confirmed that the findings of this study are highly reliable and stable (see Appendix).

## 4 Discussion

This study systematically examined the relationship between DA and adolescent SWB via a meta-analysis of 17 empirical studies (*N* = 30,915, age range 10–25 years), providing a quantitative synthesis of both the strength and the variability of this association across different samples and DA subtypes. This study finds that DA significantly negatively affects adolescent SWB (overall effect size = −0.30, 95% CI [−0.34, −0.27]), consistent with previous research (Afroz, 2016; Yang et al., 2016). This pooled effect size corresponds approximately to an r of about −0.30, indicating a moderate negative association between DA and SWB. Specifically, adolescents with DA report significantly lower life satisfaction and positive emotional experiences than those with no or low levels of DA, often accompanied by higher levels of negative emotions. These findings further confirm that excessive digital use harms adolescents’ SWB. Moreover, subgroup analyses showed that this negative association was observed across all DA subtypes and both younger (<18 years) and older (≥18 years) adolescents, with overlapping confidence intervals (Table 2), suggesting that the harmful association between DA and SWB is robust across different forms of digital engagement and developmental stages. Notably, the magnitude of this impact is comparable to that of common psychological issues such as depression and anxiety, suggesting that digital addiction is a significant risk factor for adolescent mental health (Marciano et al., 2022; Mohamadian et al., 2020).

In the primary analysis we pooled effect sizes across different subtypes of DA (internet, gaming, smartphone, and social media addiction). This decision was grounded in conceptualizations of DA as an umbrella construct that captures a common pattern of compulsive and poorly controlled digital engagement across platforms (Basel et al., 2020; Christakis, 2019). Although the specific content of use differs (e.g., online gaming vs. social networking), these subtypes share core features such as salience, tolerance, withdrawal, and functional impairment, and they are hypothesized to influence adolescents’ psychosocial adjustment through overlapping mechanisms (e.g., displacement of offline activities, sleep disruption, and social comparison). Consistent with this perspective, our subgroup analyses suggested that the magnitude of the negative association with SWB was comparable across DA subtypes, with overlapping confidence intervals (Table 2), supporting the treatment of DA as a higher-order construct in the main model. The findings of this study are partially supported by established theories. According to Self-Determination Theory, individuals have three fundamental psychological needs: autonomy, competence, and relatedness (Ryan and Deci, 2022). However, DA undermines these three fundamental psychological needs in adolescents’ real lives. For example, the algorithm-driven “information cocoon” limits autonomous choice, while the “instant feedback” of digital products contrasts with the “delayed gratification” of real life, and the false sense of competence fosters a tendency to escape reality. Furthermore, “shallow interactions” in virtual socializing cannot replace the deep emotional connections of real relationships, exacerbating feelings of disconnection and lack of belonging. Social Support Theory, conversely, emphasizes that emotional, material, and informational support from social networks such as family, school, and peers is crucial for maintaining mental health and enhancing SWB (Sarason, 2013; Vaux, 1988). Adolescents with addiction often provoke parent–child conflicts due to excessive internet use, weakening family emotional support. Additionally, declining academic performance and social alienation hinder their ability to gain attention from teachers and recognition from peers. Moreover, negative digital factors, such as cyberbullying and harmful content, intensify psychological stress. This explains why DA consistently diminishes adolescents’ SWB.

The substantial heterogeneity observed in this meta-analysis (*I*^2^ = 86.6%) implies that the true DA-SWB association varies considerably across studies and contexts. The subgroup analyses were not sufficient to account for this variability. Given the limited number of included studies, we did not perform formal meta-regression analyses, as such models would likely yield unstable or weak estimates. Consequently, the unexplained heterogeneity may reflect unmeasured factors, such as differences in measurement instruments, cultural contexts, or the conceptualization of DA and SWB. Although the direction of the effect was consistently negative and sensitivity analyses indicated that no single study unduly influenced the pooled estimate, the large between-study variance cautions against over-interpreting the exact magnitude of the average effect size. In practical terms, our findings suggest that while DA is reliably associated with lower subjective well-being among adolescents, the strength of this association can range from small to relatively large depending on contextual and methodological factors. Therefore, the overall estimate should be viewed as a summary of a distribution of effects rather than a universal parameter that applies equally to all youth populations.

One plausible source of the observed heterogeneity lies in differences in the instruments used to assess both DA and SWB. The included studies relied on multiple DA scales and diverse wellbeing measures. These instruments differ in their conceptual focus, response formats, and cut-off criteria, which may lead to non-equivalent effect size estimates even when applied to similar populations. Moreover, samples varied in terms of age composition, gender balance, and educational context, and studies were conducted in distinct cultural and temporal contexts, all of which may shape both digital use patterns and subjective wellbeing. Future research should explicitly examine measurement invariance and reliability across DA and SWB instruments, and consider harmonizing key constructs to reduce measurement-induced heterogeneity.

To mitigate the negative impact of DA on adolescents’ SWB, comprehensive intervention strategies should be developed at multiple levels: family, school, society, and the individual. Given that the negative DA-SWB association was robust across DA subtypes and age groups but varied by region and study characteristics, such interventions should be both multi-level and context-sensitive. At the family level, parents should adopt a balanced approach, establishing family internet rules, increasing parent–child interactions, and promoting a healthy digital environment, such as by implementing a “Family No Internet Day” to encourage face-to-face communication (Canadian Paediatric Society and Health, 2019; Dowdell, 2011). These strategies directly target the mechanisms highlighted in this meta-analysis, including displacement of offline activities and erosion of family support. Schools should incorporate digital health literacy into their curricula, fostering critical thinking and time management skills, implementing psychological screening to provide personalized counseling for at-risk students, and offering diverse campus activities to promote genuine achievement (Hyman et al., 2020; Schulenkorf et al., 2021). By strengthening students’ self-regulation and providing alternative sources of competence and relatedness, school-based programs may help buffer the detrimental impact of DA on SWB. Societal-level interventions should include stronger internet regulation and anti-addiction systems, the development of educational products by internet companies, and the organization of offline, interest-based activities by communities to engage adolescents during their leisure time (Chen, 2009; Science and Telecommunications Board, 1994). At the individual level, adolescents should focus on self-education to develop healthy digital usage habits, cultivate genuine interests and hobbies, and improve self-control, ultimately enhancing their SWB. In combination, these multi-level interventions align with the present meta-analytic evidence that DA constitutes a meaningful and modifiable risk factor for adolescents’ subjective well-being.

## 5 Limitations and future studies

This study has several limitations. Firstly, the study primarily utilizes cross-sectional designs, which limit the ability to establish causal relationships between DA and SWB. This makes it difficult to determine whether addiction causes a decline in well-being or if low well-being drives adolescents to rely on digital devices, raising concerns about reverse causality and confounding factors. Secondly, the measurement tools lack standardization, with substantial variations in the definitions of DA and the dimensions used to assess SWB across studies. This undermines the comparability of effect sizes and compromises the accuracy of the findings. Thirdly, the investigation of potential moderating variables is not comprehensive. While regional effects have been identified, the roles of gender, cultural values, family, social, and environmental factors remain underexplored. Finally, because reliability was inconsistently reported and item-level data were unavailable, we could not formally test measurement invariance across instruments and cultures; thus, residual heterogeneity may partly reflect measurement non-equivalence.

Future research could focus on the following areas. Firstly, studies should employ longitudinal tracking designs or experimental interventions combined with neuroscientific techniques, such as functional magnetic resonance imaging, to dynamically monitor the bidirectional mechanisms between DA and SWB, thereby clarifying causal relationships. Secondly, efforts should focus on establishing unified measurement standards that integrate core indicators of DA and SWB, and developing standardized assessment tools adaptable to diverse cultural contexts, thereby enhancing the generalizability of research findings. Finally, expanding the research perspective to examine the interactions among various environmental factors, such as family, school, and society, would provide a theoretical foundation for the development of targeted intervention strategies.

## 6 Conclusion

This study employed a meta-analysis to systematically examine the impact of DA on adolescent SWB. The results revealed a significant negative correlation between DA and SWB. DA negatively impacts SWB through multiple pathways, including displacing time spent on real-life activities, increasing feelings of loneliness, fostering cognitive biases, and disrupting family and social support networks. Therefore, developing a collaborative intervention framework involving family, school, society, and individual efforts is crucial.

## Data Availability

The original contributions presented in the study are included in the article/Supplementary material, further inquiries can be directed to the corresponding author.
