# Supplementary material for: The influence of digital addiction on adolescents’ subjective wellbeing: a meta-analysis
Source: Front Psychol. 2026 Mar 5;17:1776619. doi: 10.3389/fpsyg.2026.1776619 (PMC12999806; doi:10.3389/fpsyg.2026.1776619)

**Appendix A.**Literature Search

**PudMed**

#1 (Adolescent [MeSH Terms]) OR (Adolescents [Title/Abstract]) OR (Adolescence[Title/Abstract]) OR (Teens[Title/Abstract]) OR (Teen [Title/Abstract]) OR (Teenagers[Title/Abstract]) OR (Teenager[Title/Abstract]) OR (Youth[Title/Abstract]) OR (Youths [Title/Abstract]) OR (Adolescents, Female [Title/Abstract]) OR (Adolescent, Female[Title/Abstract]) OR (Female Adolescent [Title/Abstract]) OR (Female Adolescents [Title/Abstract]) OR (Adolescents, Male[Title/Abstract]) OR (Adolescent, Male[Title/Abstract]) OR (Male Adolescent[Title/Abstract]) OR (Male Adolescents[Title/Abstract])

#2 (Subjective Happiness[Title/Abstract] OR Happiness[Title/Abstract] OR Well-being[Title/Abstract] OR Life satisfaction[Title/Abstract])

#3 (Internet Addiction Disorders [MeSH Terms]) OR (Addiction Disorder, Internet [Title/Abstract]) OR (Addiction Disorders, Internet [Title/Abstract]) OR (Disorder, Internet Addiction [Title/Abstract]) OR (Disorders, Internet Addiction [Title/Abstract]) OR (Internet Addiction Disorders [Title/Abstract]) OR (Internet Addiction[Title/Abstract]) OR (Addiction, Internet[Title/Abstract]) OR (Addictions, Internet[Title/Abstract]) OR (Internet Addictions[Title/Abstract]) OR (Social Media Addiction [Title/Abstract]) OR (Addiction, Social Media [Title/Abstract]) OR (Addictions, Social Media [Title/Abstract]) OR (Media Addiction, Social [Title/Abstract]) OR (Media Addictions, Social [Title/Abstract]) OR (Social Media Addictions[Title/Abstract]) OR (Smartphone Addiction[Title/Abstract]) OR (Addiction, Smartphone[Title/Abstract]) OR (Addictions, Smartphone[Title/Abstract]) OR (Smartphone Addictions[Title/Abstract]) OR (Internet Gaming Disorder [Title/Abstract]) OR (Disorder, Internet Gaming [Title/Abstract]) OR (Disorders, Internet Gaming [Title/Abstract]) OR (Gaming Disorder, Internet [Title/Abstract]) OR (Gaming Disorders, Internet [Title/Abstract]) OR (Internet Gaming Disorders [Title/Abstract]) OR (Digital Addiction [Title/Abstract])

#1 AND #2 AND #3

**Web of Science**

(Adolescent OR Adolescents OR Adolescence OR Teens OR Teen OR Teenagers OR Teenager OR Youth OR Youths OR Adolescents, Female OR Adolescent, Female OR Female Adolescent OR Female Adolescents OR Adolescents, Male OR Adolescent, Male OR Male Adolescent OR Male Adolescents) (Topic) AND (Subjective Happiness OR Happiness OR Well-being OR Life Satisfaction) (Topic) AND (Internet Addiction Disorders OR Addiction Disorder, Internet OR Addiction Disorders, Internet OR Disorder, Internet Addiction OR Disorders, Internet Addiction OR Internet Addiction Disorders OR Internet Addiction OR Addiction, Internet OR Addictions, Internet OR Internet Addictions OR Social Media Addiction OR Addiction, Social Media OR Addictions, Social Media OR Media Addiction, Social OR Media Addictions, Social OR Social Media Addictions OR Smartphone Addiction OR Addiction, Smartphone OR Addictions, Smartphone OR Smartphone Addictions OR Internet Gaming Disorder OR Disorder, Internet Gaming OR Disorders, Internet Gaming OR Gaming Disorder, Internet OR Gaming Disorders, Internet OR Internet Gaming Disorders OR Digital Addiction) (Topic)

**Embase**

#1 'adolescent'/exp OR 'adolescent' OR 'adolescents'/exp OR 'adolescents' OR 'adolescence'/exp OR 'adolescence' OR 'teens' OR 'teen' OR 'teenagers' OR 'teenager'/exp OR 'teenager' OR 'youth'/exp OR 'youth' OR 'youths' OR 'adolescents, female' OR 'adolescent, female' OR 'female adolescent' OR 'female adolescents' OR 'adolescents, male' OR 'adolescent, male' OR 'male adolescent' OR 'male adolescents'

#2 'subjective happiness' OR 'happiness'/exp OR 'happiness' OR 'well-being'/exp OR 'well-being' OR 'life satisfaction'/exp OR 'life satisfaction'

#3 'addiction disorder, internet' OR 'addiction disorders, internet' OR 'disorder, internet addiction' OR 'disorders, internet addiction' OR 'internet addiction disorders' OR 'internet addiction'/exp OR 'internet addiction' OR 'addiction, internet'/exp OR 'addiction, internet' OR 'addictions, internet' OR 'internet addictions' OR 'social media addiction'/exp OR 'social media addiction' OR 'addiction, social media' OR 'addictions, social media' OR 'media addiction, social' OR 'media addictions, social' OR 'social media addictions' OR 'smartphone addiction'/exp OR 'smartphone addiction' OR 'addiction, smartphone' OR 'addictions, smartphone' OR 'smartphone addictions' OR 'internet gaming disorder'/exp OR 'internet gaming disorder' OR 'disorder, internet gaming' OR 'disorders, internet gaming' OR 'gaming disorder, internet' OR 'gaming disorders, internet' OR 'internet gaming disorders' OR 'digital addiction'/exp OR 'digital addiction'

#1 AND #2 AND #3

**Cochrane Library**

(Adolescent OR Adolescents OR Adolescence OR Teens OR Teen OR Teenagers OR Teenager OR Youth OR Youths OR Adolescents, Female OR Adolescent, Female OR Female Adolescent OR Female Adolescents OR Adolescents, Male OR Adolescent, Male OR Male Adolescent OR Male Adolescents) in Title Abstract Keyword AND (Subjective Happiness OR Happiness OR Well-being OR Life Satisfaction) in Title Abstract Keyword AND (Internet Addiction Disorders OR Addiction Disorder, Internet OR Addiction Disorders, Internet OR Disorder, Internet Addiction OR Disorders, Internet Addiction OR Internet Addiction Disorders OR Internet Addiction OR Addiction, Internet OR Addictions, Internet OR Internet Addictions OR Social Media Addiction OR Addiction, Social Media OR Addictions, Social Media OR Media Addiction, Social OR Media Addictions, Social OR Social Media Addictions OR Smartphone Addiction OR Addiction, Smartphone OR Addictions, Smartphone OR Smartphone Addictions OR Internet Gaming Disorder OR Disorder, Internet Gaming OR Disorders, Internet Gaming OR Gaming Disorder, Internet OR Gaming Disorders, Internet OR Internet Gaming Disorders OR Digital Addiction) in Title Abstract Keyword

**EBSCO**

(Adolescent OR Adolescents OR Adolescence OR Teens OR Teen OR Teenagers OR Teenager OR Youth OR Youths OR Adolescents, Female OR Adolescent, Female OR Female Adolescent OR Female Adolescents OR Adolescents, Male OR Adolescent, Male OR Male Adolescent OR Male Adolescents) AND (Subjective Happiness OR Happiness OR Well-being OR Life Satisfaction) AND (Internet Addiction Disorders OR Addiction Disorder, Internet OR Addiction Disorders, Internet OR Disorder, Internet Addiction OR Disorders, Internet Addiction OR Internet Addiction Disorders OR Internet Addiction OR Addiction, Internet OR Addictions, Internet OR Internet Addictions OR Social Media Addiction OR Addiction, Social Media OR Addictions, Social Media OR Media Addiction, Social OR Media Addictions, Social OR Social Media Addictions OR Smartphone Addiction OR Addiction, Smartphone OR Addictions, Smartphone OR Smartphone Addictions OR Internet Gaming Disorder OR Disorder, Internet Gaming OR Disorders, Internet Gaming OR Gaming Disorder, Internet OR Gaming Disorders, Internet OR Internet Gaming Disorders OR digital addiction)

**CNKI**

(主题:青少年)AND(主题:主观幸福感)AND(主题:数字成瘾 + 网络成瘾 + 互联网成瘾 +网络游戏成瘾 + 手机成瘾 + 社交媒体成瘾)

**WanFang**

主题:(青少年) and 主题:(主观幸福感) and 主题:(数字成瘾 or 社交媒体成瘾 or 手机成瘾 or 网络成瘾 or 互联网成瘾 or 网络游戏成瘾)

**VIP**

任意字段=青少年 AND 任意字段=主观幸福感 AND 任意字段=（数字成瘾 or 社交媒体成瘾 or 手机成瘾 or 网络成瘾 or 互联网成瘾 or 网络游戏成瘾）

**Appendix B.**Quality evaluation of the eligible studies with JBI Critical Appraisal Checklist for Analytical Cross-Sectional Studies

| Study | Were the criteria for inclusion in the sample clearly defined? | Were the study subjects and the setting described in detail? | Was the exposure measured in a valid and reliable way? | Were objective, standard criteria used for measurement of the condition? | Were confounding factors identified? | Were strategies to deal with confounding factors stated? | Were the outcomes measured in a valid and reliable way? | Was appropriate statistical analysis used? | Total |
| --- | --- | --- | --- | --- | --- | --- | --- | --- | --- |
| Afroz et al.(2016) | * | - | * | * | - | - | ○ | * | 4 |
| Cheng et al.(2023) | * | * | * | * | * | * | * | * | 8 |
| Ding et al.(2024) | * | * | * | * | * | * | * | * | 8 |
| Huang et al.(2023) | * | - | * | * | * | * | * | * | 7 |
| Islam et al.(2025) | * | * | * | * | * | * | * | * | 8 |
| Li, Ma et al.(2021) | * | * | * | * | * | * | * | * | 8 |
| Li, Tan et al.(2022) | * | * | * | * | * | * | * | * | 8 |
| Lin et al. (2023) | * | * | * | * | * | * | * | * | 8 |
| Liu et al.(2024) | * | * | * | * | * | * | * | * | 8 |
| Mahmid et al.(2021) | * | * | * | * | - | - | * | * | 6 |
| Mei et al.(2015) | * | * | * | * | - | - | * | * | 6 |
| Odaci et al.(2014) | * | * | * | * | * | * | * | * | 8 |
| Rastegarian et al. (2022) | * | * | * | * | * | * | * | * | 8 |
| Sun et al.(2022) | * | * | * | * | * | * | * | * | 8 |
| Uysal et al.(2013) | * | * | * | * | - | - | * | * | 6 |
| Ye et al.(2022) | * | * | * | * | * | - | * | * | 7 |
| Zhou et al.(2022) | * | * | * | * | * | * | * | * | 8 |

* indicates Yes; - indicates No; ○ indicates Unclear.

**Appendix C.**Quality evaluation of the eligible studies with Newcastle-Ottawa Scale

| **Study** | **Selection** | | | | **Comparability** | | **Outcome** | | |
| --- | --- | --- | --- | --- | --- | --- | --- | --- | --- |
|  | Representative-ness | Selection of non-exposed | Ascertainment of exposure | Outcome not present at start | Comparability on most important factors | Comparability on other risk factors | Assessment of outcome | Long enough follow-up(median≥1 year) | Adequacy  (completeness of follow-up) |
| Narita et al. | * | * | - | * | * | * | - | * | * |
| Raudsepp | * | * | - | * | * | * | - | * | - |
| Lung et al. | * | * | - | * | * | * | - | * | - |
| Ma et al. | * | * | - | * | * | * | - | * | - |
| Deng et al. | * | * | - | * | * | * | - | * | * |
| Chang et al. | * | * | - | * | * | * | - | * | * |
| Rudolf et al. | * | * | - | * | * | * | - | * | * |
| Zhu et al. | * | * | - | * | * | * | - | * | * |

*indicates criterion met;-indicates significant of criterion not met

**Appendix D.**Results of the Trim and Fill Method


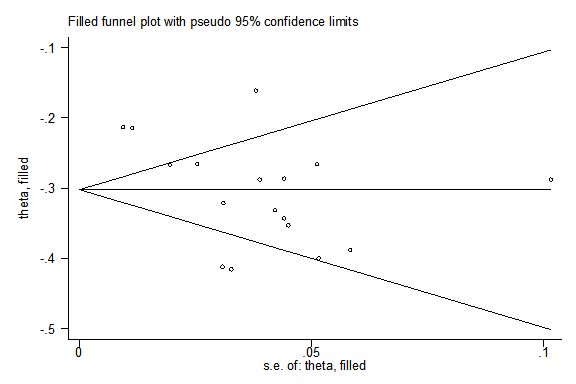

Supplement: Supplementary file 1 [file Table_1.docx]
